# On the Cause of the Death of Children When the Umbilical Cord Is Compressed during Labour

**Published:** 1787

**Authors:** John Clarke

**Affiliations:** Licentiate in Midwifery of the Royal College of Physicians, and Teacher of Midwifery in London


### XII. *On the Cause of the Death of Children when the Umbilical Cord is compressed during Labour. By* John Clarke, *Licentiate in Midwifery of the Royal College of Physicians, and Teacher of Midwifery in London.*

IT is no very uncommon thing to see cases where children, which had certainly been alive in the beginning of labour, are born dead; a circumstance which most commonly arises from compression of the funis umbilicalis. This accident may be produced in different ways; but is most frequently met with where the cord is unusually long. When this is the case, as it may lie at any part, so it may happen to be near the os uteri, and may descend, as an original presentation, into the cavity of the pelvis before any part of the child; when, if it be not returned, or the labour expedited, the child is often born dead.

It sometimes happens also, that practitioners, with a view to increase the action of the uterus, and shorten the duration of a labour, rupture the membranes, and discharge the waters, or they break spontaneously in the early part of a labour. By this means a sort of eddy or current rent is made, and the funis (which would not otherwiſe have happened) is brought by the ſtream of water through the os uteri, where, if it be ſuffered to remain, it will probably undergo compreſſion in the progreſs of the labour, and the child will be deſtroyed *.

Various opinions have been entertained as to the cauſe of the child's death from this accident, and the moſt general of theſe have been, that it was to be attributed either to apoplexy, or to the heart and veſſels being ſo diſtended

* Perhaps one great uſe of the membranes, containing the waters, may be to preſerve the life of the child from the injuries to which it might otherwiſe be liable from the contractions of the uterus; for if the funis ſhould be in an unfavourable ſituation, as in the fold of a limb, or even between the body of the child and the ſides of the uterus, the circulation through its veſſels might be endangered. This is prevented, in a great meaſure, whilſt the membranes continue whole, becauſe then the uterus does not act upon the child itſelf, but only in the ſame manner as preſſure upon a bladder, full of water, affects any thing contained within it; and accordingly, the life of the child is generally ſafe till the waters have run off. This ſhould afford a practical obſervation, viz. that we ſhould be very careful never wantonly, or from aukwardneſs in our examinations, or from a deſire to ſave our own time, to rupture the membranes when the cord might ſlip down; but by all means to avoid it, except where the ſafety of the mother or child, or both, may demand it.

with with blood, that they were rendered incapable of action, or to the courfe of nourifhment being fhut up. But in the former cafe the death would not be fo inftantaneous; and the next fuppofition is probably not founded in fact, fince at what period foever the obftruction takes place, there will only be its own proper quantity of blood in the body of the child, and therefore we can conceive it poffible that the circulation through it might be performed, if there were no other obftacle *.

But as I am difpofed to believe that the placenta certainly performs, in the fœtal ftate, a fimilar office to that which the lungs do afterwards, befides the conveyance of nourifhment, I am inclined to think that the death of children, from compreffion of the funis, is owing to want of air: that they are deftroyed in the fame manner as drowned perfons; and this will explain why the fame means of recovery will fucceed generally alike in both cafes.

The application or admiffion of air to their blood feems to be neceffary in all animals; but

* To the laft opinion it may be anfwered, that a child will live without nourifhment for fome days after birth, and therefore would not be fo foon killed for want of it in its paffage through the pelvis.

the mode in which this is effected is various. In ſome it is performed by the immediate application of air to the coats of the pulmonary veſſels, as in ſuch animals who live in the open air; in others, through the medium of fluids (capable of being impregnated with air) applied to their lungs, as in many of the tribe of ſhell fiſhes, which, from their ſtructure, are incapable of locomotion, as oyſters, &c.

If then the influence of air be neceſſary for ſupporting animal life generally, it would ſeem highly probable that it is ſo alſo to the fœtus; and upon this principle we find in oviparous animals a particular apparatus in the conſtruction of the egg for admitting air to the blood of the embryo, and another for expoſure of the blood to the air.

In viviparous animals there appears to be no other mode of effecting this than by means of the placenta.

In the conſtruction of the vaſcular ſyſtem in adults, great care is taken by nature to return the blood, which has performed the circulation, quickly to the lungs, and from thence to the heart, when it has acquired the benefit of reſpiration, ſo that it may again paſs over the whole body.

 A ſimi-

A ſimilar apparatus is found in the fœtal ſtate for carrying the blood to the placenta, and then back to the general circulation, avoiding, however, the lungs, from paſſing through which no advantage could be derived.

If in adults the admiſſion of air to the lungs be obſtructed, or excluded either by tying the trachea, or by immerſing the mouth in water, or if there be any obſtruction in the veſſels to the paſſage of the blood through them, life will be carried on with difficulty, or entirely extinguiſhed.

In like manner the powers of life will be depreſſed, or altogether loſt, by any obſtruction to the paſſage of blood through the placenta, and nearly in the ſame time as obſtruction to the paſſage of air through the lungs would deſtroy an adult. The way in which the influence of air is conveyed by the placenta to the fœtal blood probably reſembles that by which it is conveyed to oyſters, &c. by means of water; and as it is ſaid that fiſhes cannot live in water from which all the atmoſpheric air is taken away, ſo neither can a child ſurvive any accident which prevents its blood, in the very minute veſſels of the fœtal portion of the placenta, from being applied to the mother's blood in the cells of the maternal portion of the placenta, which blood being ſupplied by the ſpermatic and hypogaſtric arteries, has conſequently received the benefit of air from the lungs of the mother.

If then the defect of air be the cauſe of the death when the funis is compreſſed, the mode of recovery will reſemble that in drowned perſons.

The application of ſtimuli ſimply to drowned perſons only reproduces life by throwing the organs of reſpiration into action; and no ſtimulus has ſo powerful an effect as that of freſh air applied to the lungs. Warmth, either excited by friction, warm bath, or any other means, may alſo aſſiſt by ſupplying the body with extraneous heat, and ſo preventing the exertion of the weakened powers of life, (which otherwiſe might be exhauſted in the attempt) to reproduce and keep up its own heat; but warmth alone will not be ſufficient to revive the patient.

When children are born apparently dead, the ſame means become neceſſary for their recovery; and although we may aſſiſt the revival by warmth or friction, yet, unleſs we can produce the action of reſpiration, as the ſource of air from the placenta is at an end, all our attempts will be fruitlefs; as I had occafion very lately to lament, in a cafe which I faw along with Dr. Denman, where, though by imitating refpiration, the action of the heart was excited and kept up for an hour, yet as the mufcles of refpiration in the child were not capable of excitement, the heart at length ceafed to act, and the child died.

As it appears, then, that there is a neceffity of air for fupporting all animal life, we may conclude that it is alfo neceffary in the fœtus, and the opinion is ftrengthened by analogy with the fœtal ftate of oviparous animals.

2. By comparing the return of the blood to the placenta in the fœtus with that to the lungs in adults, it appears probable that their offices are fimilar.

3. If the former pofitions be true, then the confequences of any obftruction to the circulation in the adult, through the trachea or the pulmonary veffels, and in the fœtus, through the funis umbilicalis, will be analogous; and

4. The means of recovery (viz. the application of the influence of air to the blood again) will be the fame in both inftances, which we find to be the cafe.

5. There-

5. Therefore I think we may fairly deduce this inference, that children, which are deftroyed by compreffion of the funis umbilicalis, die for want of thofe advantages which animals receive from the influence of air upon their blood.

*Chancery Lane,*
May 31, 1787.

